# [^18^F]SPA-RQ/PET Study of NK1 receptors in the Whole Body of Guinea Pig and Rat

**DOI:** 10.1038/s41598-019-56848-3

**Published:** 2019-12-31

**Authors:** Tove J. Grönroos, Sarita Forsback, Olli Eskola, Jörgen Bergman, Päivi Marjamäki, Eliisa Löyttyniemi, Jarmo Hietala, Merja Haaparanta-Solin, Olof Solin

**Affiliations:** 1 Turku PET Centre, University of Turku, Turku, Finland; 2 Medicity Research Laboratory, University of Turku, Turku, Finland; 3 Department of Chemistry, University of Turku, Turku, Finland; 4 Department of Biostatistics, University of Turku, Turku, Finland; 5 Department of Psychiatry, University of Turku and Turku University Hospital, Turku, Finland; 6 Accelerator Laboratory, Åbo Akademi University, Turku, Finland

**Keywords:** Target validation, Experimental models of disease

## Abstract

There is a substantial interest in the development of NK1 substance P antagonists as potential treatments for various neuropsychiatric and somatic disorders. The aim of this study was to determine whether [^18^F]SPA-RQ can be utilized as a tool for studying the whole body distribution and function of NK1 receptors in preclinical settings. The compound was injected into guinea pigs with or without premedication with a NK1 receptor antagonist (NK1A-2). For comparison, we included two rats in the study, as the affinity of antagonists for NK1 receptors is known to vary between species. The whole body biodistribution of the tracer was determined at several time points. The tracer showed specific binding in organs compatible with the known location of NK1-receptors. Premedication with a NK1 antagonist led to an inhibited uptake of [^18^F]SPA-RQ in several organs of guinea pigs, notably intestine, pancreas, urinary bladder, uterus, skin and lung. Specific binding was also seen in both cortex and striatum. In contrast, negligible specific binding was observed in the rat brain with [^18^F]SPA-RQ, whereas the tracer uptake in peripheral tissues was similar to that seen in guinea pigs. We conclude that [^18^F]SPA-RQ/PET is a useful tool to study the distribution and function of peripherally located NK1 receptors e.g. in different disease models.

## Introduction

The mammalian tachykinins (TKs) are a family of closely related peptides traditionally classified as neurotransmitters that includes substance P (SP), neurokinin A (NKA) and neurokinin B (NKB). Tachykinins are distributed throughout the central and peripheral nervous system and many different physiological functions, such as e.g. inflammation and smooth muscle contraction, have been linked to them^1^. The TKs mediate their effect via specific G-protein coupled receptors using predominantly the phosphoinositol system as a second messenger pathway. Three types of tachykinin receptors, NK1, NK2 and NK3 are heterogeneously distributed within species. SP is the natural endogenous ligand of NK1 receptors while NKA and NKB are the preferential ligands of NK2 and NK3 receptors, respectively^2^.

For many years, the tachykinins were considered almost exclusively as peptides of neuronal origin. However, the concept has changed due to evidence showing that TKs can be produced also by non-neuronal cells such as immune and inflammatory cells^3,4^. Moreover, an advance in the field of tachykinins has been the discovery of three new mammalian TKs, hemokinin-1 (HK1) and its human orthologs, endokinin A (EKA) and endokinin B (EKB)^5–7^. A common feature for HK1, EKA and EKB is that they are all primarily expressed in non-neuronal cells and they have shown a high selectivity for the NK1 receptor^8,9^.

SP is by far the best characterized TK and on the basis of anatomical localization and functional studies there is considerable interest in the development of NK1 substance P antagonists (SPAs) for treatments of a variety of disorders. Such disease processes includes inflammation, pain, affective and addictive disorders, as well as functional disorders of the intestine and urine bladder^1^.

Clinical trials with a NK1 receptor antagonist, aprepitant (Emend^®^), has proven to be an effective antiemetic drug in humans and this drug is now available for the treatment of chemotherapy-induced nausea and vomiting^10,11^. Aprepitant was not effective in the treatment of depression but has shown antitumor activity and proven to be effective for the therapy of itch^12–15^. Indeed, overexpression of NK1 receptors has been implicated in cancer progression and poor overall prognosis^16^. Research for new targets continues including receptors in peripheral regions such as urinary and gastrointestinal tracts.

We have previously reported on the synthesis of fluorine-18 labelled SPA-RQ ([2-[^18^F]fluoromethoxy-5-(5-trifluoromethyl-tetrazol-1-yl)-benzyl]([2*S*,3*S*]2-phenyl-piperidin-3-yl)-amine, [^18^F]SPA-RQ), a selective NK1 receptor antagonist with a very high affinity for this receptor^17^. Preliminary studies showed that [^18^F]SPA-RQ selectively bound to NK1 receptors in guinea pig (GP) brain and later it has shown to be a useful tracer for *in vivo* imaging of human brain NK1 receptors with PET^17–22^. The radiation dosimetry of [^18^F]SPA-RQ in human has also been reported^23^.

We here report the specific binding of [^18^F]SPA-RQ in the whole body of GPs and rats as to provide a tool to study the function and expression of NK1 receptors outside the brain. The structure of TK receptors, as well as the affinity of non-peptide antagonists for NK1 receptors is known to vary between species^24,25^. The receptors in the human resembles those in GP and therefore GP is an ideal species to study the function of TKs. For comparison, we also include rats in this study.

An *ex vivo* experimental PET study approach enables to investigate NK1 receptors at higher spatial resolution and sensitivity than in an *in vivo* setting. Furthermore, we illustrate the power of the *ex vivo* autoradiography methodology for determining the intra-organ distribution of the tracer. To define the specific binding of [^18^F]SPA-RQ the biodistribution was also determined in GPs premedicated with a selective NK1 antagonist.

## Results

### Synthesis of [^18^F]SPA-RQ

**Figure 1:**
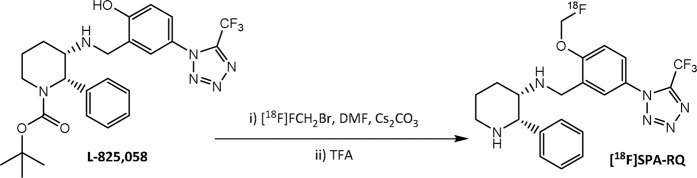
Radiochemical synthesis of [^18^F]SPA-RQ. [^18^F]FCH_2_Br = [^18^F]bromofluoromethane, Cs_2_CO_3_ = cesium carbonate, DMF = dimethylformamide, TFA = trifluoroacetic acid.

The radiotracer [^18^F]SPA-RQ was synthesized via O-fluoromethylation of the L-825,058 precursor in DMF (Fig. 1). The synthesis route yielded [^18^F]SPA-RQ at very high molar activity, ranging from 180 to 2480 GBq/µmol, with a mean value of 920 GBq/µmol at the end of synthesis. The radiochemical purity exceeded 98.5% in all cases.

### Peripheral biodistribution of [^18^F]SPA-RQ

**Figure 2:**
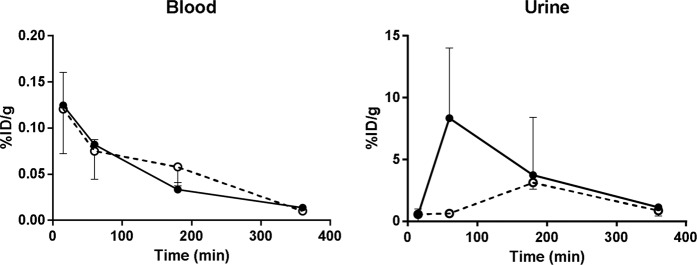
^18^F-radioactivity uptake in blood and urine as a function of time. Data originating from nonmedicated GPs are shown in black symbols and premedicated in white symbols (dashed line). Data in all groups are for three animals per time point and treatment, if not otherwise stated in the Supplementary Table 1. Note that the y-scale (% ID/g) varies between the tissues.

The peripheral biodistribution results of [^18^F]SPA-RQ from GPs that were either nonmedicated or premedicated with the NK1 selective antagonist are shown in Supplemental Table 1. The uptake of the tracer from blood into tissues was fast (Fig. 2). [^18^F]SPA-RQ and its radioactive metabolites were moderately bound to plasma proteins. Of the total ^18^F-radioactivity, free radioactivity in GP plasma was 35–41% at 15 min after the [^18^F]SPA-RQ injection. The fraction of free ^18^F-radioactivity increased slightly over time, being 46% and 51% at 60 and 180 min after the injection, respectively.

**Figure 3:**
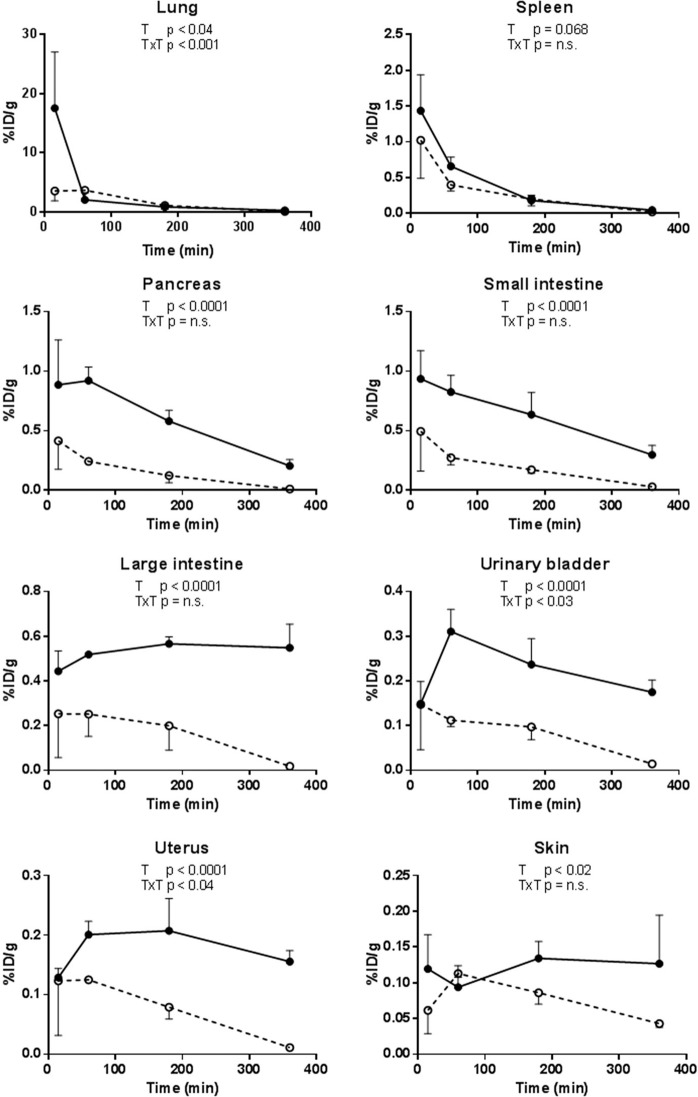
^18^F-radioactivity uptake in selected organs from nonmedicated (black symbols), and GPs premedicated (2 mg/kg) with a NK1 antagonist (white symbols, dashed line) as a function of time. Data in all groups are for three animals per time point and treatment. Note that the y-scale (% ID/g) varies between the different tissues. ANOVA: T, treatment effect; T x T, time x treatment interaction.

**Figure 4:**
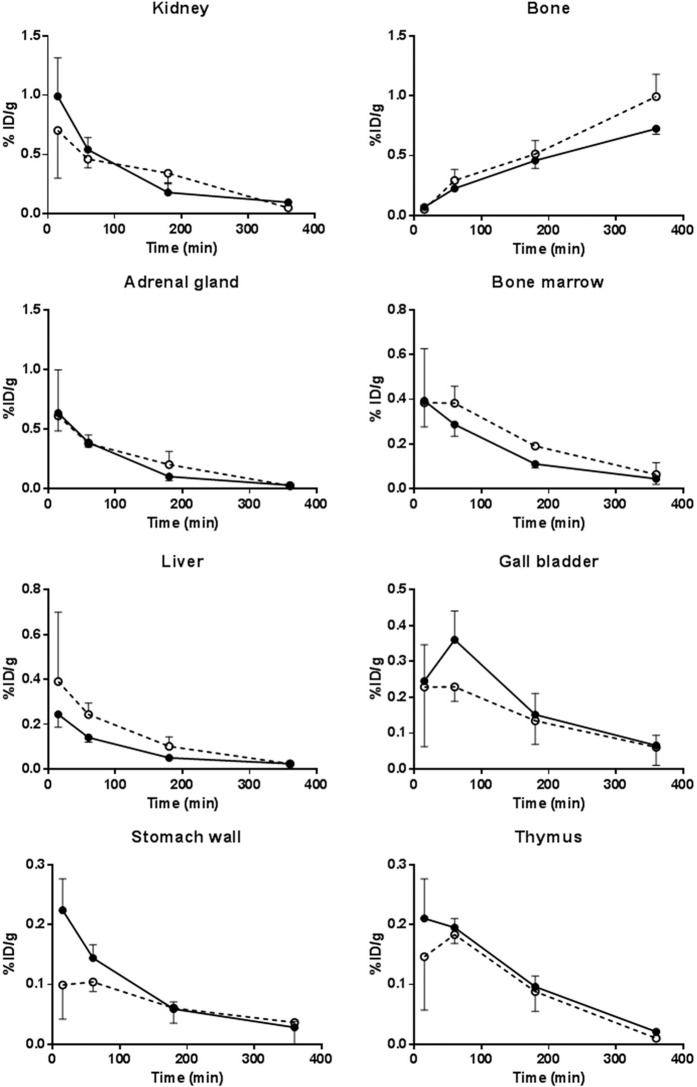
^18^F-radioactivity uptake in selected organs unaffected by the NK1 antagonist pretreatment (2 mg/kg) as a function of time. Organs originating from nonmedicated GPs are shown in black symbols and premedicated in white symbols (dashed line). Data in all groups are for three animals per time point and treatment, if not otherwise stated in the Supplementary Table 1. Note that the y-scale (% ID/g) varies between the different tissues. ANOVA: T, treatment effect; T × T, time × treatment interaction.

Up to 60 min post injection the highest ^18^F-radioactivity uptakes were seen in lungs, pancreas, small intestine, spleen, and kidneys (Figs. 3 and 4). At 360 min post injection high uptake of the tracer was still seen in lungs, pancreas and small intestine. Throughout the study high uptake of the tracer was also seen in the large intestine, urinary bladder, uterus, skin, urine and bone. The tracer was primarily excreted through the urinary pathway as a high amount of the tracer was measured from the urine (Fig. 2). Increasing amounts of ^18^F-radioactivity was measured in the bone (parietal bone in the skull) as a function of time, indicating that some defluorination of the tracer occurred, as earlier reported^17^.

Several tissues were clearly affected by the treatment (NK1A-2; 2 mg/kg) (Fig. 3). A clear effect on the ^18^F-radioactivity uptake was seen in small intestine, large intestine, pancreas, uterus, urinary bladder and skin. Furthermore, a moderate effect was seen in lung and a trend toward an effect was detected in the spleen.

**Figure 5:**
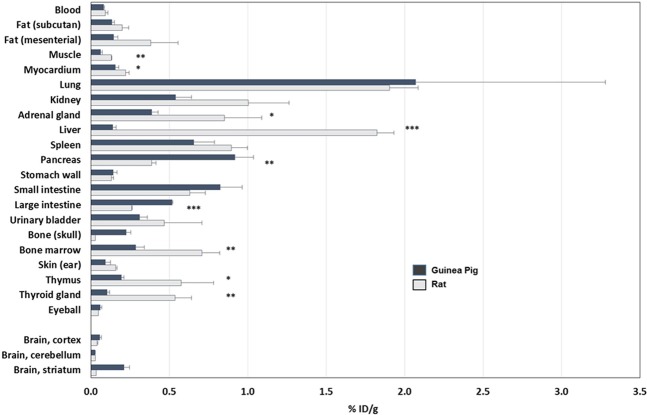
^18^F-radioactivity uptake in GP and rat tissues at 60 min post injection of [^18^F]SPA-RQ. Values are from three GPs and two rats (rat striatum, muscle and eyeball; n = 1). Significances are marked as *p < 0.05, **p < 0.01 and ***p < 0.001.

The biodistribution results of [^18^F]SPA-RQ from rats are shown in Supplemental Table 2. The uptake pattern of [^18^F]SPA-RQ in tissues of rats compared to GPs is shown in Fig. 5. A significantly higher uptake was seen in the rat heart (p < 0.05), liver (p < 0.001), bone marrow (p < 0.01), adrenal glands (p < 0.05), thymus (p < 0.05) and thyroid glands (p < 0.01), whereas a significantly lower uptake was detected in the pancreas (p < 0.01) and large intestine (p < 0.001). The rat kidney, spleen, fat and urinary bladder showed a trend toward a significantly higher uptake in the rats compared to GPs.

### Regional brain distribution

**Figure 6:**
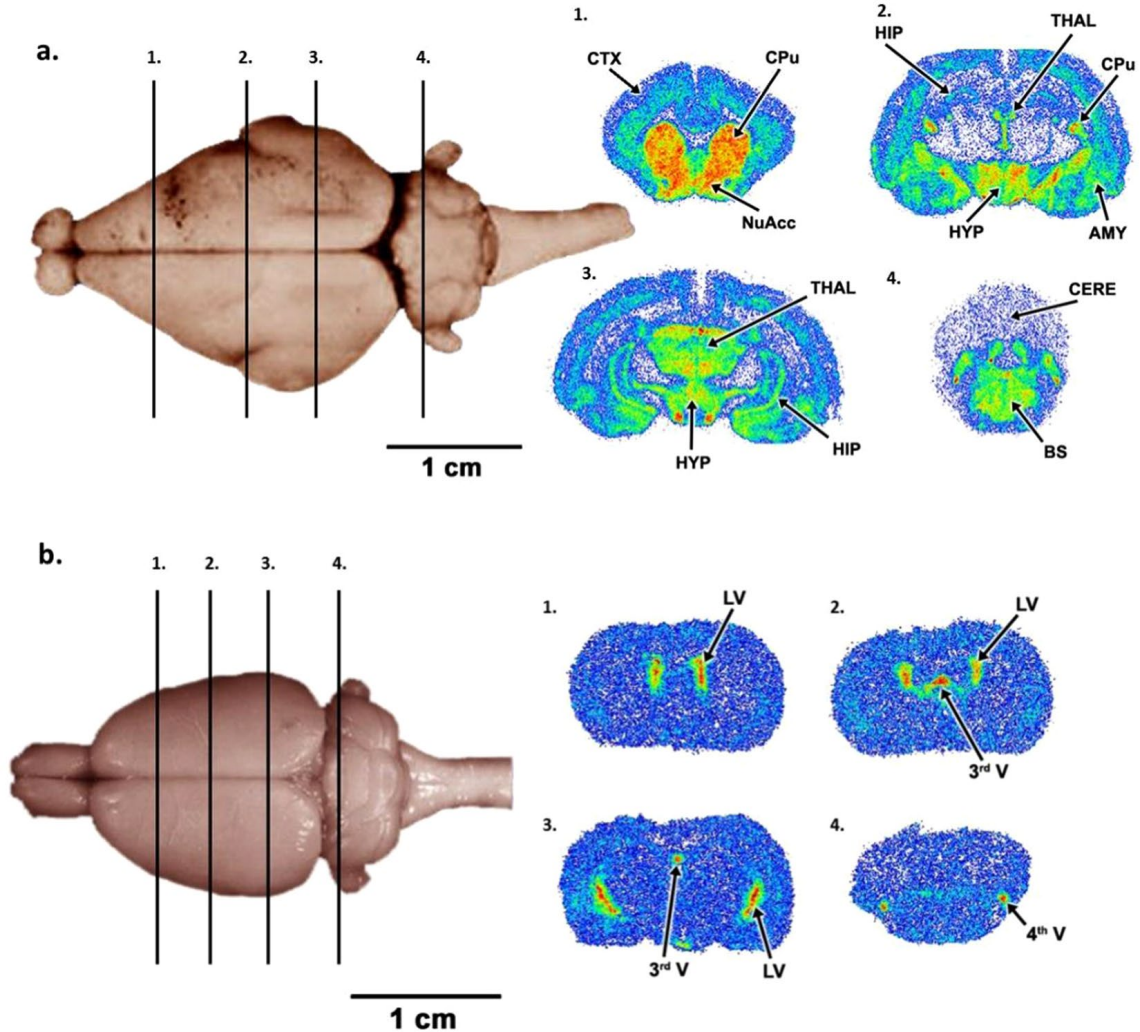
Uptake of [^18^F]SPA-RQ in (**a**) GP and **b**) rat brain. The numbered (1–4) bars (on the left) indicate the position from where the four brain slices shown at right were cut. The regional biodistribution of [^18^F]SPA-RQ in coronal slices of the GP brain at 180 min post injection is shown on the upper right (AMY = amygdala, BS = brainstem, CERE = cerebellum CPu = caudate-putamen, CTX = cortex, HIP = hippocampus, HYP = hypothalamus, NuAcc = nucleus accumbens and THAL = thalamus). The regional biodistribution of [^18^F]SPA-RQ in coronal slices of the rat brain at 60 min post injection is shown on the lower right (LV = lateral ventricle, 3^rd^ V = 3^rd^ ventricle, 4^th^ V = 4^th^ ventricle).

A typical example of the uptake of [^18^F]SPA-RQ into GP brain at 180 min post injection is shown in Fig. 6a. Nonmedicated animals showed fast and high uptake of the tracer in the caudate/putamen region as well as a very low uptake in the cerebellum. [^18^F]SPA-RQ also showed high uptakes in regions of thalamus, hypothalamus and midbrain regions. Uptake of ^18^F-radioactivity was also seen in the cortex (Fig. 6a). [^18^F]SPA-RQ uptakes in different brain regions from nonmedicated and premedicated GPs expressed as % ID/g tissue calculated from the biodistribution data are shown in Supplemental Table 1. The uptake of [^18^F]SPA-RQ into rat brain is shown in Fig. 6b. High ^18^F-radioactivity uptake was seen in lateral ventricle, 3^rd^ ventricle and 4^th^ ventricle and a clear lack of uptake was seen in the areas of caudate-putamen, cortex, thalamus and hypothalamus. Lack of specific binding in the caudate/putamen (striatum) region was also seen in the biodistribution data (Fig. 5).

## Discussion

TKs represent one of the largest known peptide families and can be found in different animal species from low invertebrates to mammals. The TKs and their receptors have been highly conserved throughout evolution, which argues for an important biological function that is probably still only partly understood^2^. They are widely distributed throughout the mammalian body, being primarily distributed in the central and peripheral nervous system but also in non-neuronal cells. In neuronal cells tachykinins acts as neurotransmitters or neuromodulators, whereas they function as autocrine, paracrine or endocrine regulators in non- neuronal cells.

As the NK1 receptor is a potential treatment target for a variety of disorders, we evaluated [^18^F]SPA-RQ as a tool to investigate the whole body expression and function of NK1 receptors. It has been reported that there are many-fold inter-species differences in the activity of non-peptide NK1 ligands^26,27^. In order to visualize the inter-species differences in the NK1 receptor distribution, two rats were also studied. Very weak [^18^F]SPA-RQ specific binding to NK1 receptors in the rat CNS was indeed detected.

Autoradiographic studies using [^125^I]SP as a NK1 probe have shown high binding in the amygdala, nucleus accumbens, septum, caudate, putamen, hippocampus, hypothalamus, locus coeruleus, substantia nigra, and the frontal, entorhinal, and olfactory cortices in monkey and human^28^. In line with these findings, we and others have previously reported on a high affinity and selective binding of [^18^F]SPA-RQ to NK1 receptors in GP and human brain^17–22,28^. However, in the current study we demonstrate the usage of [^18^F]SPA-RQ for imaging peripherally located NK1 receptors.

In peripheral neuronal cells, tachykinins can have sensory or motor functions. The source of tachykinins in the peripheral nervous system is a population of sensory nerves, which are activated by capsaicin^29,30^. When stimulated, a number of neuropeptide transmitters, including SP and NKA are released, which initiate functional responses.

Our results with GPs show that the peripheral uptake of [^18^F]SPA-RQ is in good agreement with the known biodistribution of SP and NK1 receptors. The highest ^18^F-radioactivity uptake in the periphery was seen in the lung. SP has been suggested to be a sensory neuropeptide in the lung, where it increases vascular permeability and produces smooth muscle constriction^31^. Recently, it has become clear that neuropeptides, including SP, are released not only from nerve endings but also from inflammatory immune cells, which affect nervous or immune cells in a paracrine or autocrine fashion^32–34^. It has also been reported that the immune and nervous systems together optimizes defence systems within the lung^35^. The uptake of [^18^F]SPA-RQ was especially high in the lungs at 15 min post injection and continuously high uptake was seen throughout the study, even though the uptake did decrease as a function of time. In premedicated GPs this uptake was significantly affected (p < 0.04) as shown in Fig. 3. This effect was probably mainly due to the apparently reduced ^18^F-radioactivity uptake at 15 min post injection in premedicated GPs, meanwhile it did slightly increase at 60 and 180 min after injection of [^18^F]SPA-RQ. This might be due to the complex role of SP, acting both as a neurotransmitter and an autocrine regulator in the lung.

^18^F-radioactivity uptake of [^18^F]SPA-RQ in organs of the immune system was measured in bone marrow, thymus and spleen. At early time points, a quite high uptake was seen in the spleen. This uptake decreased as a function of time and significantly reduced uptake (p < 0.001) in premedicated animals was only seen at 360 min post injection. The overall treatment effect did, however, show a trend toward significance (p = 0.068).

We also found, in agreement with the known function of SP, a high ^18^F-radioactivity uptake in both small- and large intestine. The uptake in the both intestines behaved quite similar and was significantly (p < 0.0001) decreased in premedicated GPs. In the intestine SP is believed to be released into the gut by neurons found in the sympathetic and parasympathetic autonomic nervous system as well as in the enteric nervous system. The released SP will activate cells which participate in the control of gastro-intestinal motility, secretion, circulation and tissue homoeostasis^36^. A high ^18^F-radioactivity uptake was also seen in the pancreas. This uptake was also significantly decreased (p < 0.0001) in premedicated GPs. SP released from the primary sensory fibers has been identified as one of the key neurotransmitters in pancreas. Receptors for SP have been detected on pancreatic acinar cells where they stimulate enzyme secretion^37^. SP did also promote proliferation of pancreatic ductal cells but not their differentiation into β-cells^38^, and preserved pancreatic β-cells in type 1 and type 2 diabetic mice^39^. Other gastro-intestinal tissues measured were the stomach wall, liver and the gall bladder. None of these tissues showed high accumulation of [^18^F]SPA-RQ, nor could the ^18^F-radioactivity uptake be blocked by the NK1 antagonist, although SP containing nerve fibers has been located in periportal and intralobular regions in the liver of different species (including GP) and in bile duct smooth muscle of GP^40^.

A moderate ^18^F-radioactivity uptake could also be detected in the urinary bladder wall, uterus and skin. Our results indicate a NK1 receptor specific uptake in these organs, since the uptake was significantly reduced by the NK1 antagonist. Sensory nerves in the guinea pig bladder wall are known to contain SP^41^. The precise function of SP as a neurotransmitter in the bladder wall and the uterus is unknown, but it has been suggested to contribute to physiological mechanisms such as the contraction of the bladder and uterus^42,43^. Sensory nerves in the skin control the vascular permeability and SP is also believed to be involved in neurogenic inflammations of skin^44^.

Even though a lack of uptake in the brain was detected in rats, the tracer showed quite similar peripheral tissue uptakes compared to GPs. The limited number of rats included in this study does, however, most probably affects our results to some degree. Generally, most studies have focused on developing new antagonist for NK1 receptors in the brain and hence the rat, as a NK1 receptor model, is less used. Our preliminary results does, however, indicate that [^18^F]SPA-RQ can be used for studying the function of peripheral NK1 receptors in the rat, as well.

In summary, PET imaging of NK1 receptors in peripheral organs will be useful in studies resolving the role of NK1 receptors in pathophysiological processes and diseases as well as in drug development. Based on our findings NK1 receptor imaging in peripheral tissues could be used e.g. for imaging of inflammatory processes^33^ in the gastrointestinal tract (IBD)^36^, respiratory system (asthma, chronic bronchitis)^4^ and pancreas (pancreatitis)^45^ providing possible targets for therapeutic interventions. Even though not evaluated in the current study, imaging of NK1 receptors in cancer is also an attractive field of application^16,46^.

Applying NK1 imaging to human peripheral tissues might need further characterization of tissue specific pharmacodynamic properties of the tracer, especially if aiming for full quantitation of receptor characteristics in proof-of concept type studies or NK1 receptor occupancy for dose-finding studies in drug development. However, for some applications semi-quantitative measures might be sufficient and easily translated to human applications.

## Conclusions

Our results in GPs demonstrate that the uptake of [^18^F]SPA-RQ is consistent with earlier localization of NK1 receptors and indicate that [^18^F]SPA-RQ can be useful for studying the expression and function of peripherally located NK1 receptors e.g. in different disease models.

## Materials and Methods

### Synthesis of [^18^F]SPA-RQ

High molar activity [^18^F]bromofluoromethane was used as the labelling reagent. After the removal of DMF, hydrolysis was carried out in TFA and [^18^F]SPA-RQ was purified with semi-preparative HPLC. More detailed descriptions of the synthesis of [^18^F]bromofluoromethane and the synthesis of [^18^F]SPA-RQ can be found in literature^17,47^.

### Animals

Female GP (Mol:Dunkin-Hartley, Möllegaard Breeding & Research Centre A/S, Skensved, Denmark) and male rats (Harlan Sprague-Dawley, Central Animal Laboratory, University of Turku, Turku, Finland) were used in this study. The animals were housed under standard conditions (temperature 21 °C; humidity 55 ± 5%; 12 h light/dark cycle) with free access to standard food and tap water. Animals were cared for in accordance with the Directives 2012/707/EU, 2014/11/EU and of the European Parliament and of the Council for the Care and Use of Laboratory Animals. Licenses were obtained from the Regional State Administrative Agencies in Finland.

### Biodistribution study

Altogether twenty four GPs (530 ± 29 g) and two rats (295 ± 7 g) were used. GPs were anesthetized with i.m. injections of Ketamine/Xylazine (Ketamine 30 mg/kg, Xylazine 5 mg/kg) given prior to the administration of the tracer. In order to occupy the target receptors, twelve GPs were premedicated (2 mg/kg) with NK1A-2, a potent brain penetrant NK1 selective antagonist, in 10% ethanol/saline 5 min before the injection of [^18^F]SPA-RQ^48^. The tracer and the antagonist were injected through a cannula inserted in the jugularis. The injected doses varied depending on the distribution time and were 6.4 ± 2.6 MBq, 14 ± 5.1 MBq, 48 ± 26 MBq and 82 ± 19 MBq for GPs studied at 15, 60, 180 and 360 minutes, respectively. In rats, the tracer was administered (35 ± 25.2 MBq) via a tail vein and studied at 60 min post injection without pretreatment. The administrated amounts of non-radioactive SPA-RQ, calculated from the injected radioactivity and from the molar activity of [^18^F]SPA-RQ, varied from 20 to 200 ng. At each time point three nonmedicated and three premedicated GPs were studied. The animals were sacrificed and the organs of interest were immediately dissected and measured for ^18^F-radioactivity in a well counter (3 × 3 inches NaI (Tl) crystal, Bicron 3MW3/3 P, Bicron Inc., Newbury, Ohio, USA) and weighed. Brains were also rapidly removed, frozen in isopentane chilled with dry ice and prepared as described in the section on regional brain distribution. In addition, pieces of brain tissue from cortex, cerebellum and striatum from each animal were measured separately for absolute radioactivity. All data were corrected for background radioactivity and radioactivity decay. The uptake of ^18^F-radioactivity in tissues was expressed as percentage of injected dose per gram tissue weight (% ID/g).

### Plasma protein binding

Cardiac blood samples were collected from GPs at 15, 60 and 180 min after [^18^F]SPA-RQ injection into gel-lithium heparin tubes (Terumo Europe, Leuven, Belgium), and plasma was separated by centrifugation (1 300 g, 10 min). The protein free fraction was separated from plasma by filtration with Filtron Microsep 30 K ultrafiltration tubes (3 030 g, 2 × 15 min; Filtron Technology Corporation, Northborough, MA) and the amount of radioactivity was measured before and after filtration to assess the free fraction of ^18^F-radioactivity in plasma.

### Regional brain distribution

The regional brain distribution of [^18^F]SPA-RQ was studied using a digital autoradiography technique (Fuji BAS 5000, Fuji Photo Film Co. Ltd. Japan) in one nonmedicated GP and one rat. This technique has the advantages of being very sensitive for detecting the β^+^ particles emitted in the decay of ^18^F as well as having comparatively good spatial resolution (pixel size 25 × 25 µm). Serial coronal brain sections (20 μm) were obtained using a cryomicrotome. The brain sections were thaw-mounted onto microscope slides, air dried and apposed to an imaging plate (Fuji Imaging Plate BAS-TR2025, Fuji Photo Film Co., Ltd., Japan). The exposure time was 4 ± 0.5 h. The imaging plates were then scanned with the Fuji Analyzer BAS-5000. Regions were anatomically identified from the sections using a GP brain atlas^49^.

### Statistics

Data are given as mean ± standard deviation (SD). Two-way analysis of variance (ANOVA) was used with the factors treatment (nonmedicated, premedicated) and time (15, 60, 180 and 360 min after injection of [^18^F]SPA-RQ) and their interaction. Square-root- and log-transformations were used to meet assumptions of ANOVA. If the interaction was not significant it was removed from analysis and only main effects were included in the final model. For simplicity, mean ± SD are used in figures. The analyses were performed with SAS System software, version 9.3 for Windows (SAS Institute, Cary, NC, USA). Differences in [^18^F]SPA-RQ uptake between GPs and rats were tested with an unpaired t-test and calculations carried out with use of GraphPad Prism software v. 6.07 (GraphPad Software, San Diego, CA, USA). Differences were considered significant if p < 0.05 (two-tailed).

## Supplementary information

Supplementary information.
