# Supplementary material for: [18F]SPA-RQ/PET Study of NK1 receptors in the Whole Body of Guinea Pig and Rat
Source: Sci Rep. 2019 Dec 31;9:20412. doi: 10.1038/s41598-019-56848-3 (PMC6938475; doi:10.1038/s41598-019-56848-3)
Supplement: Supplementary file 1 — Supplementary information. [file 41598_2019_56848_MOESM1_ESM.pdf]

Electronic Supplementary Material

[^{18}F]SPA-RQ/PET Study of NK1 receptors in the Whole Body of Guinea Pig and Rat

Tove J. Grönroos[1,2], Sarita Forsback[1,3], Olli Eskola[1], Jörgen Bergman[1], Päivi Marjamäki[1], Eliisa Löyttyniemi[4], Jarmo Hietala[1,5], Merja Haaparanta-Solin[1,2], Olof Solin[1,3,6]

[1] Turku PET Centre, University of Turku, Turku, Finland
[2] Medicity Research Laboratory, University of Turku, Turku, Finland
[3] Department of Chemistry, University of Turku, Turku, Finland
[4] Department of Biostatistics, University of Turku, Finland
[5] Department of Psychiatry, Turku University Hospital, Turku, Finland
[6] Accelerator Laboratory, Åbo Akademi University, Turku, Finland

Table 1. Biodistribution of [^{18}F]SPA-RQ in nonmedicated (nonmed) or in guinea pigs premedicated (premed) with a NK1 selective antagonist. Data in all groups are mean ± SD %ID/g of three animals.

Tissue	15 min		60 min		180 min		360 min	
	Nonmed	Premed	Nonmed	Premed	Nonmed	Premed	Nonmed	Premed
Blood	0.12 ± 0.04	0.12 ± 0.05	0.08 ± 0.01	0.08 ± 0.03	0.03 ± 0.01	0.06 ± 0.02	0.01 ± 0.00	0.01 ± 0.00
Fat (subcutan)	0.12 ± 0.05	0.09 ± 0.05	0.14 ± 0.01	0.17 ± 0.01	0.07 ± 0.02	0.11 ± 0.05	0.02 ± 0.00	0.02 ± 0.01
Fat (mesenterial)	0.18 ± 0.08	0.11 ± 0.06	0.15 ± 0.03	0.19 ± 0.02	0.08 ± 0.01	0.11 ± 0.02	0.02 ± 0.00	0.01 ± 0.00
Muscle	0.06 ± 0.01	0.05 ± 0.04	0.06 ± 0.01	0.08 ± 0.01	0.02 ± 0.01	0.04 ± 0.02	0.01 ± 0.00	0.01 ± 0.00
Myocardium	0.38 ± 0.14	0.31 ± 0.13	0.16 ± 0.02	0.17 ± 0.01	0.06 ± 0.02	0.08 ± 0.03	0.02 ± 0.00	0.01 ± 0.00
Lung	18 ± 9	3.6 ± 1.7	2.0 ± 1.2	3.7 ± 1.8	0.85 ± 0.23	1.1 ± 0.67	0.29 ± 0.04	0.11 ± 0.03
Kidney	0.99 ± 0.32	0.70 ± 0.40	0.54 ± 0.10	0.46 ± 0.07	0.18 ± 0.07	0.34 ± 0.08	0.10 ± 0.03[a]	0.05 ± 0.00[a]
Adrenal gland	0.64 ± 0.15	0.61 ± 0.39	0.39 ± 0.04	0.38 ± 0.07	0.10 ± 0.04	0.20 ± 0.11	0.03 ± 0.01	0.03 ± 0.01
Liver	0.24 ± 0.06	0.39 ± 0.31	0.14 ± 0.02	0.24 ± 0.05	0.05 ± 0.01	0.10 ± 0.04	0.02 ± 0.00	0.02 ± 0.01
Spleen	1.4 ± 0.51	1.0 ± 0.53	0.66 ± 0.13	0.40 ± 0.08	0.18 ± 0.07	0.20 ± 0.09	0.05 ± 0.01	0.02 ± 0.01
Pancreas	0.89 ± 0.38	0.42 ± 0.24	0.92 ± 0.12	0.24 ± 0.01	0.58 ± 0.09	0.12 ± 0.06	0.21 ± 0.05	0.01 ± 0.01
Gall bladder	0.25 ± 0.10	0.23 ± 0.17	0.36 ± 0.08	0.23 ± 0.04	0.15 ± 0.06	0.14 ± 0.07	0.07 ± 0.03	0.06 ± 0.05
Stomach (wall)	0.22 ± 0.05	0.10 ± 0.06	0.14 ± 0.02	0.10 ± 0.02	0.06 ± 0.01	0.06 ± 0.02	0.03 ± 0.01	0.04 ± 0.04
Small intestine	0.94 ± 0.24	0.50 ± 0.33	0.82 ± 0.14	0.28 ± 0.06	0.64 ± 0.19	0.17 ± 0.03	0.30 ± 0.08	0.03 ± 0.01
Large intestine	0.44 ± 0.09	0.25 ± 0.20	0.52 ± 0.01	0.25 ± 0.10	0.57 ± 0.03	0.20 ± 0.11	0.55 ± 0.11	0.02 ± 0.00
Urine	0.52 ± 0.46	0.57 ± 0.78	8.3 ± 5.7	0.63 ± 0.07	3.7 ± 4.7[a]	3.1 ± 0.5	1.2 ± 0.2	0.85 ± 0.42
Urinary bladder	0.15 ± 0.05	0.15 ± 0.10	0.31 ± 0.05	0.11 ± 0.01	0.24 ± 0.06	0.10 ± 0.03	0.18 ± 0.03	0.01 ± 0.00
Uterus	0.13 ± 0.02	0.12 ± 0.09	0.20 ± 0.02	0.12 ± 0.01	0.21 ± 0.05	0.08 ± 0.02	0.16 ± 0.02	0.01 ± 0.00
Bone (skull)	0.07 ± 0.02	0.05 ± 0.03	0.23 ± 0.03	0.29 ± 0.09	0.46 ± 0.07	0.52 ± 0.11	0.73 ± 0.05	0.99 ± 0.19
Bone marrow	0.39 ± 0.12	0.38 ± 0.24	0.29 ± 0.05	0.38 ± 0.08	0.11 ± 0.02	0.19 ± 0.02	0.04 ± 0.02	0.06 ± 0.05
Skin	0.12 ± 0.05	0.06 ± 0.03	0.09 ± 0.03	0.11 ± 0.01	0.13 ± 0.02	0.09 ± 0.02	0.13 ± 0.07	0.04 ± 0.01
Thymus	0.21 ± 0.07	0.15 ± 0.09	0.20 ± 0.02	0.18 ± 0.02	0.10 ± 0.02	0.09 ± 0.03	0.02 ± 0.00[a]	0.01 ± 0.00
Thyroid gland	0.15 ± 0.04	0.12 ± 0.08	0.10 ± 0.02	0.14 ± 0.02	0.05 ± 0.01	0.06 ± 0.02	0.02 ± 0.01	0.01 ± 0.00
Eyeball	0.08 ± 0.02	0.03 ± 0.02	0.06 ± 0.01	0.03 ± 0.00	0.04 ± 0.01	0.02 ± 0.01	0.03 ± 0.00	0.01 ± 0.00
Cortex	0.07 ± 0.04	0.05 ± 0.03	0.06 ± 0.01	0.03 ± 0.00	0.03 ± 0.01	0.01 ± 0.01	0.01 ± 0.00	0.01 ± 0.00
Striatum	0.18 ± 0.05[b]	0.05 ± 0.04	0.21 ± 0.03[b]	0.03 ± 0.00	0.21 ± 0.05[b]	0.01 ± 0.01	0.10 ± 0.02[b]	0.01 ± 0.00
Cerebellum	0.06 ± 0.02	0.05 ± 0.04	0.03 ± 0.00	0.03 ± 0.00	0.01 ± 0.00	0.02 ± 0.00	0.01 ± 0.00	0.01 ± 0.00

[a] n = 2, [b] Data achieved from autoradiography study.

Table 2. Biodistribution of [^{18}F]SPA-RQ in nonmedicated rat. Data are mean ± SD %ID/g of two animals.

Tissue	60 min
Blood	0.09 ± 0.02
Fat (subcutan)	0.20 ± 0.04
Fat (mesenterial)	0.38 ± 0.18
Muscle	0.13 ± n.a.[a]
Myocardium	0.22 ± 0.02
Lung	1.9 ± 0.2
Kidney	1.0 ± 0.26
Adrenal gland	0.85 ± 0.24
Liver	1.8 ± 0.11
Spleen	0.90 ± 0.10
Pancreas	0.39 ± 0.03
Gall bladder	n.a.
Stomach (wall)	0.13 ± 0.01
Small intestine	0.63 ± 0.10
Large intestine	0.21 ± 0.01
Urine	3.8 ± 1.6
Urinary bladder	0.47 ± 0.24
Testes	0.08 ± 0.01
Bone (skull)	0.39 ± 0.10
Bone marrow	0.71 ± 0.11
Skin	0.16 ± 0.01
Thymus	0.57 ± 0.21
Thyroid gland	0.54 ± 0.10
Eyeball	0.05 ± n.a.[a]
Cortex	0.04 ± 0.01
Striatum	0.03 ± n.a.[a]
Cerebellum	0.03 ± 0.00

n.a. = not available, [a] n = 1
